# Association of breakfast styles such as Japanese, Western, and cereals with sleeping habits, eating habits, and lifestyle in preschool and elementary school children

**DOI:** 10.3389/fnut.2023.1131887

**Published:** 2023-06-30

**Authors:** Mai Kuwahara, Yu Tahara, Lyie Nitta, Akiko Furutani, Seiko Mochida, Naomichi Makino, Yuki Nozawa, Shigenobu Shibata

**Affiliations:** ^1^Laboratory of Physiology and Pharmacology, School of Advanced Science and Engineering, Waseda University, Tokyo, Japan; ^2^Graduate School of Biomedical and Health Sciences, Hiroshima University, Hiroshima, Japan; ^3^Benesse Education Research and Development Institute, Benesse Corporation, Okayama, Japan

**Keywords:** Japanese breakfast, Western breakfast, cereal, sleep, eating habits, protein sources, morning type, evening type

## Abstract

**Introduction:**

In Japan, breakfast styles are categorized into five groups; Japanese breakfast (JB; rice and miso soup), Western breakfast (WB; bread and milk), Japanese-Western breakfast (J-WB; alternative daily serving), cereal breakfast (CB), and breakfast skipping. In our recent studies, breakfast style was highly associated with the daily sleep–wake phase (chronotype), and healthy eating habits. In contrast with other breakfast style consumers, JB-consumers were positively associated with the morning chronotype and healthy eating habits such as a high consumption of a variety of protein sources, vegetables, and dietary fibers, and low consumption of sweetened juices. These previous studies included only adult participants; hence, in the current study, we investigated whether similar observations can be made in children.

**Methods:**

Preschool (aged 3–5 years) and elementary school children (6–8 years) (N = 6,104, 49.87% boys, 50.13% girls, mean body mass index 15.39 ± 0.03 kg/m^2^ for preschoolers and percentage of overweight −2.73 ± 0.22 for elementary school children) participated in this cross-sectional online survey on lifestyle, including eating and sleep habits, through their mother’s responses.

**Results:**

The results showed that the morning-evening type index values (chronotype indicator, smaller indicates morning type) were negatively correlated with JB intake (−0.05, *p* < 0.01) and positively correlated with WB (0.03, *p* < 0.05) and CB intake (0.06, *p* < 0.01), suggesting that the JB group exhibited the morning chronotype and the WB and CB groups exhibited the evening chronotype. The JB group consumed a variety of protein sources (mean ± SE; days/week) with more frequency (fish 2.95 ± 0.038 *p* < 0.001, soy 3.55 ± 0.043 *p* < 0.001, egg 3.82 ± 0.044 *p* < 0.001) compared with the WB group (fish 2.58 ± 0.033, soy 3.00 ± 0.038, egg 3.49 ± 0.039). On the other hand, the JB group consumed snacks (5.48 ± 0.042 *p* < 0.001) and sweetened juice (2.50 ± 0.050 *p* < 0.001) less frequently than the WB group (snacks; 5.80 ± 0.037 and sweetened juice; 2.74 ± 0.049).

**Discussion:**

JB-eating children with a morning chronotype exhibited better sleep and eating habits than WB-eating children with an evening type pattern. The results suggest that JB eating habits may be associated with good eating and sleeping lifestyles, even among preschool and elementary school children.

## 1. Introduction

Sleep has a significant impact on a healthy life. Particularly, longitudinal studies in Japan have shown that sleeping hours have decreased progressively because of the late time to fall asleep, and sleep quality has declined over the past few decades (1). However, the actual sleep state and its relationship with sleeping habits and behavior in developing children are not fully understood (2). Children’s sleeping habits are directly influenced by those of their parents, and irregular or late sleeping habits in parents are associated with children’s sleep disturbances, daytime sleepiness, and irregular eating habits (3). Furthermore, comparing mothers and fathers, the former’s sleeping habits had a stronger impact on children’s sleep than did the latter’s in adolescents (3, 4). A few studies have examined the association of preschool/elementary school children’s sleeping habits and behavior with those of their parents. Adolescents’ sleep patterns are correlated with those of their parents, with factors such as the adolescents’ sex, father’s level of education, and parents’ alcohol consumption frequency, particularly influencing the adolescents’ sleep duration (4, 5). These studies focused on sleep duration and quality but not on circadian rhythm-related factors such as chronotype. Sleep rhythms, such as chronotypes and social jet lags, are directly linked to the rhythms of life.

Dietary factors can influence sleep quality and rhythm (6). Individuals with morning chronotypes eat healthier than those with evening types, with a more frequent intake of fish, fruits, whole grains, and rye (7). Cross-sectional and experimental studies have demonstrated the benefits of eating earlier in the day regarding postprandial blood glucose levels. People with the evening type are driven to consume food later in the day (8). The evening type is associated with largely unhealthy eating habits associated with obesity (9, 10). Individuals with the evening type consume snacks more frequently and later at night; this chronotype shows significantly higher fasting total cholesterol and low-density lipoprotein cholesterol levels and significantly lower postprandial insulin sensitivity (11).

Breakfast is the most important meal of the day (12), and studies have shown that consuming protein sources for breakfast effectively elevates exercise habits. Examining the relationship between sleeping and eating habits can help reduce the incidence of disease and lower blood pressure and blood glucose levels. Breakfast has an immediate positive effect on children’s brain development and cognitive function (13–15).

Japanese people have a low incidence of coronary artery disease and are known for their longevity (16). This has long attracted the interest of other countries because of the possible contribution of the Japanese diet. However, certain problems are associated with the Japanese diet (17); excess salt intake through the consumption of miso soup, salted vegetables, soy sauce, and commercial seafood is an example. Despite the high sodium intake, the Japanese have an overall low incidence of cardiovascular diseases. This is probably due to the high potassium intake through vegetables. Japanese meals are characterized by high fish and soy products and low animal fats and meat consumption. Therefore, this characteristic could be a valuable tool for supporting healthy eating habits (18). In addition, foodstuffs in Japanese meals prevent the onset of diseases; frequent consumption of seaweed, vegetables, mushrooms, legumes, potatoes, and starches lowers the risk of non-alcoholic fatty liver disease in Japanese men (19) and reduces the risk of sarcopenia in Japanese men and women (20). It has also been introduced as a healthy diet for preventing COVID-19, and its effectiveness has been evaluated in scientific studies (21, 22). Furthermore, soy reduces blood pressure and glucose levels (23, 24). A recent study suggested that Japanese breakfast (JB) consumption is associated with the intake of protein sources such as fish, eggs, and soy and is beneficial for good eating habits, including a balanced diet (1). In addition, a study reported the benefits of JB on nutrition intake and physical activity (25, 26). Adults who consumed JB preferred mornings, while those who consumed Western breakfast (WB) or cereal breakfast (CB) preferred evenings (27). Many of these studies included only adults and older adults, and there are no reports examining the relationship between chronotype and breakfast style among preschool and early elementary school children. Healthy eating habits at an early age are important for supporting good eating habits throughout life. This study aimed to elucidate the relationship between breakfast style and chronotype in children.

Good eating habits are defined as having a balanced diet rich in protein sources, vegetables, and fruits, with a low consumption of snacks and sweets. Good eating habits and breakfast intake styles are related and directly connected to health (25); in particular, they correlate with physical activity levels, cardiovascular indices, and the cognitive and academic performance of young people (28). Children are also influenced by their parents’ eating habits. The body mass index (BMI) values were influenced more by the mother (29–31). Poor eating habits established in childhood can persist into adulthood and increase the risk of developing obesity and obesity-related complications such as type 2 diabetes. Modification of eating habits in childhood has been shown to improve health and reduce the risk of developing diseases later in life (30).

The primary aim of this study was to examine the effect of differences in breakfast styles (JB, J–WB [Japanese–Western breakfast], WB, and CB) on sleep parameters such as wakeup/sleep onset time, sleep duration, and chronotype of children aged 3–8 years and their parents. We also examined the intake frequency of various protein sources, vegetables, and fruits as healthy eating habits, and that of snacks and sweetened juice as unhealthy eating habits across breakfast styles. A high frequency of intake of various protein sources, vegetables, and fruits indicated good eating habits, whereas a high frequency of intake of snacks and soft drinks, such as juice, indicated unhealthy ones (25). We also aimed to identify the differences in the food intake patterns between JB with the morning chronotype as a healthy group and WB with the evening chronotype as an unhealthy group.

## 2. Materials and methods

### 2.1. Ethical approval

The Ethics Review Committee on Research Involving Human Subjects at Waseda University approved this study (No. 2021-101). In addition, the guiding principles of the Declaration of Helsinki were followed.

### 2.2. Target population and data collection

The respondents to the questionnaire items were mothers of children aged 3–8 years; the children and their parents were residents of Japan (from Hokkaido to Okinawa). An online survey company (Macromill Inc., Tokyo, Japan) was commissioned to conduct this survey. Data were collected from 6,180 people. The aforementioned company has a large pool of applicants who could respond to the survey; thus, it was relatively easy to recruit 6,180 participants. Moreover, participants could respond to the web-based survey quickly and efficiently; hence, the survey was completed within 4 days (July 1–4, 2022). Among the 6,180 respondents, three reported living outside the country and were excluded. Respondents with missing values for sleep-related variables were excluded, and 6,104 people were ultimately analyzed. Using our previous cross-sectional web-based survey on elementary and high school students, we performed a power analysis for multiple regression analysis with confounding factors to determine the sample size (32).

### 2.3. Questionnaire

The questionnaire aimed to determine the sleeping habits of children aged 3–8 years and the eating habits of their parents and siblings. The questionnaire could be completed within 20 min. All questionnaires were completed by the mothers instead of the children or their fathers. Participants who did not live with their children were excluded from this survey; we focused on mothers who lived with their children to ensure accurate responses. Four questions on basic information were asked, including the sex, age, height, and body weight of the respondent’s children, and these values were self-reported by the mothers.

Four questions on sleep parameters were asked: wake-up time on weekdays with or without an alarm, that on weekends and holidays without wake-up alarm, and sleep onset time on weekdays and weekends/holidays. Following recent papers (25–27), the breakfast style of the respondent’s children was categorized into five groups: JB, a pattern in which Japanese food items such as rice are included; J–WB, a pattern in which Japanese and Western food items are eaten alternately; WB, a pattern in which bread is included; CB, a pattern in which cereals are included; and missing breakfast (SB) (Supplementary Table 1). In Japan, the aforementioned breakfast styles were easy to understand as JB: including rice and soy sauce; WB: including bread and salad or milk products; and CB: including cereal and milk or soy milk. J–WB included alternately served JB and WB. We did not ask about the breakfast styles of the parents; however, we believe that the entire family would have taken the same breakfast.

For analyzing eating habits, there were seven questions regarding the respondents’ children’s intake frequency of food items in their usual diet (eight levels, 0–7 days/week). In the current study, the food items were categorized into three groups. The first group consisted of several questions to examine the variation in protein sources: meat, fish, eggs, soy, and dairy products (Supplementary Table 2). The second group included two questions to investigate the intake frequency of vegetables and fruits (Supplementary Table 3) to estimate healthy nutrients such as potassium and dietary fibers (33). The third group consisted of two questions examining the intake of unhealthy foods, such as snacks and soft drinks (Supplementary Table 3). As this study aimed to investigate the impact of different breakfast styles on sleep, eating habits, and lifestyle, data from participants who did not respond about their breakfast style was excluded.

MSFsc (Chronotype Index; Midpoint time of sleep time on weekdays and holidays), SJL (Social Jet Lag), and SLOSS (Sleep Deprivation Index) were used as the sleep indices. Smaller MSFsc values indicate more morning-type characteristics, and a large MSFsc value indicates more evening-type characteristics. Smaller SJL values indicate less social jet lag. SLOSS indicates the degree of sleep deprivation per week, with higher numbers indicating greater sleep deprivation. The calculations of these sleep parameters have been previously published (34).

The MSFsc can be expressed by the following equation:

$$\mathrm{MSFsc}={MSF}^{*1}-\left({SDh}^{*2}-{\mathrm{SDweek}}^{*3}\right)/2.$$

*1 Midpoint time of sleep time on weekdays and holidays.

*2 Sleep duration on holidays.

*3 Sleep duration on weekdays and holidays.

SJL can be expressed by the following equation:

SJL=Time of wakingonholidays−Time of wakingonweekdays.

Absolute values were calculated and analyzed as data.

SLOSS score can be expressed by the following equation:

$$\begin{array}{l} \mathrm{When SDweek}>{SDw}^{*4}:\mathrm{SLOSSweek}=\left(\mathrm{SDweek}-SDw\right)\;\times {WD}^{*5}.\\ \mathrm{when Sdweek}\leq SDw:\mathrm{SLOSSweek}\\ \;=\left(\mathrm{Sdweek}-SDh\right)\times \left(7-WD\right)\text{.} \end{array}$$

*4 Sleep duration on weekdays.

*5 Number of days per week in preschool or elementary school.

### 2.4. Chronotype division

The two groups, divided into morning and evening types, were divided by two near-equal numbers of children calculating MSFsc for each grade in boys and girls because MSFsc values were higher in older girls (32). The actual MSFsc limits were 2:00 AM in boys aged 3 years, 2:04 in girls aged 3 years, 2:00 in boys aged 4 years, 2:04 in girls aged 4 years, 2:04 in boys aged 5 years, 2:15 in girls aged 5 years, 2:00 in boys aged 6 years, 2:04 in girls aged 6 years, 2:08 in boys aged 7 years, 2:08 in girls aged 7 years, 2:15 in boys aged 8 years, and 2:15 in girls aged 8 years.

### 2.5. Body mass index and percentage of overweight

We calculated BMI (Kg/m^2^) for children aged 3–5 years because there are similar BMI values among 3–5 years old Japanese boys and girls (35). Conversely, BMI is not a good marker for 6–8 year-old children. Therefore, we calculated POW by adjusting age and sex using the growth curve for Japanese children (35). Thin and fat children were defined as having <13 and > 18 kg/m^2^ BMI, and < −20% and > +20% POW, respectively.

### 2.6. Statistical analysis

Statistical analyses were performed using IBM SPSS Statistics version 28 (IBM Ltd., Armonk, NY, United States). Student’s *t*-test was used for sex-based comparisons of data on children’s basic information, sleeping habits, and eating habits. Mann–Whitney U-test was used for POW Chi-square test was used for the sex-based comparison of children’s breakfast styles. Spearman’s rank correlation coefficient was used to examine the relationship of children’s breakfast style with their sleeping and eating habits. Kruskal–Wallis and Dann tests were used to compare the breakfast style and chronotype groups. Multiple regression analysis was used to examine the relationship between chronotype and usual food intake. In this case, the objective variable was the MSFsc value. The explanatory variables were meat, fish, eggs, soy, dairy products, vegetables, fruits, snacks, and juice. Adjustment factors were age, sex, and BMI. Statistical significance was set at *p* < 0.05.

## 3. Results

### 3.1. Characteristics of the target group and results of the questionnaire

Participants (*n* = 6,104) comprised 49.87% boys and 50.13% girls. The mean age was 5.50 ± 0.02 years. Comparisons between boys and girls were made using Student’s *t*-test. The JB, J–WB, WB, CB, and SB styles were followed by 2,188, 1,389, 2,300, 172, and 55 children, respectively. Comparisons between boys and girls were performed using Fisher’s exact test (Table 1). The proportion of JB boys was significantly higher than that of JB girls (Table 1). Children’s mean wake-up times were 6:37 on weekdays and 7:14 on weekends, and their mean bedtime was 21:09 on weekdays and 21:25 on weekends (Supplementary Table 4). The MSFsc, SJL, and SLOSSweek for children were 2:08, 0:29, and 0:56 h, respectively (Supplementary Table 4). Comparisons between boys and girls were made using Student’s *t*-test (Supplementary Table 4). Holiday wake-up time, holiday sleep length, MSFsc, SJL, and SLOSS values were significantly higher in girls than in boys (Supplementary Table 4). The frequency of weekly intake of meat, fish, eggs, soy, dairy products, vegetables, fruit, sweets, and juices is presented in Supplementary Table 5. The intake frequencies of meat (*p* < 0.05), dairy products (*p* < 0.001), and snacks (*p* < 0.05) were significantly higher in boys than in girls. In contrast, the intake frequency of vegetables (*p* < 0.05) was significantly higher in girls than in boys.

**Table 1:** Basic information of participants.

Category	ALL (*n* = 6,104)	Boys (*n* = 3,044)	Girls (*n* = 3,060)	*p* value Boys vs. Girls
Age [years]	5.50 ± 0.02	5.50 ± 0.03	5.50 ± 0.03	0.95^*1^
BMI [kg/m^2^]	15.18 ± 0.03	15.27 ± 0.05	15.09 ± 0.05	*p* < 0.01^*1^
POW [%]	−2.73 ± 0.22	−2.74 ± 0.35	−2.61 ± 0.37	0.65^*2^
JB [people]	2,188	1,139	1,049	*p* < 0.05^*3^
J-W B [people]	1,389	664	725	0.08^*3^
WB [people]	2,300	1,123	1,177	0.21^*3^
CB [people]	172	85	87	0.91^*3^
SB [people]	55	33	22	0.13^*3^

Data are expressed as mean ± SE. ^*1^Student’s *t*-test was used. ^*2^Man-Whitney U test was used. ^*3^Chi-square test was used.

### 3.2. Relationship between breakfast style and sleep habits

**Figure 1:**
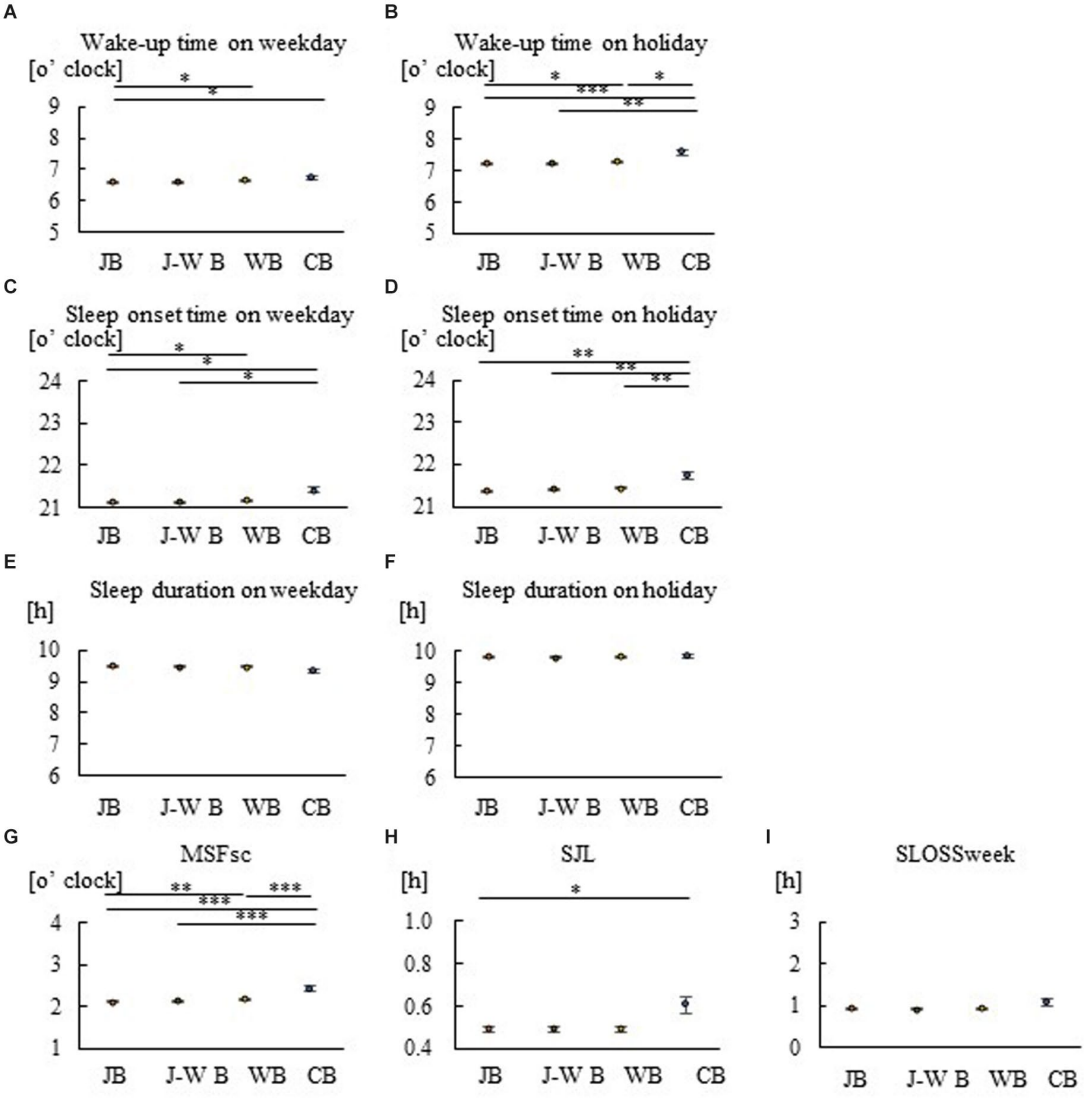
Group comparison of breakfast styles for sleep in children. Wake-up time on weekdays **(A)**, wake-up time on holidays **(B)**, sleep onset time on weekdays **(C)**, sleep onset time on holidays **(D)**, sleep duration on weekdays **(E)**, sleep duration on holidays **(F)**, MSFsc **(G)**, SJL **(H)**, and SLOSS **(I)**. Consumption of JB leads to earlier waking and sleeping times on weekdays and holidays, more preference for morning hours, and less social jetlag. Consumption of WB or CB, on the other hand, results in later waking and sleeping times, longer evening hours, and more social jet lag. *p < 0.05, **p < 0.005, ***p < 0.001. A Kruskal–Wallis test with Bonferroni correction was used.

**Figure 2:**
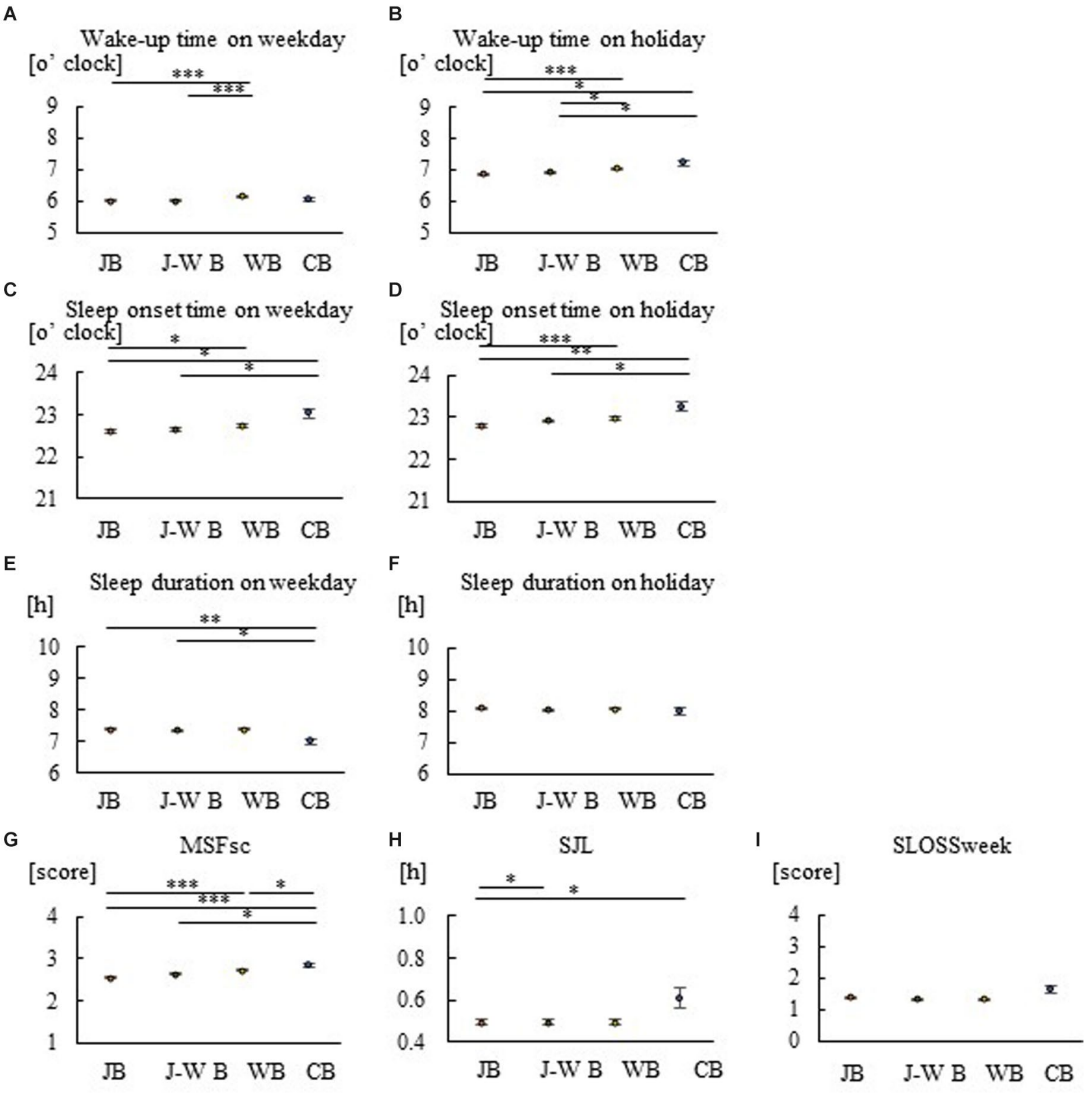
Group comparison of breakfast styles for sleep in mothers. Wake-up time on weekdays **(A)**, wake-up time on holidays **(B)**, sleep onset time on weekdays **(C)**, sleep onset time on holidays **(D)**, sleep duration on weekdays **(E)**, sleep duration on holidays **(F)**, MSFsc **(G)**, SJL **(H)**, and SLOSS **(I)**. Consumption of JB leads to earlier waking and sleeping times on weekdays and holidays, more preference for morning hours, and less social jetlag. Consumption of WB or CB, on the other hand, results in later waking and sleeping times, longer evening hours, and more social jet lag. *p < 0.05, **p < 0.005, ***p < 0.001. A Kruskal–Wallis test with Bonferroni correction was used.

**Figure 3:**
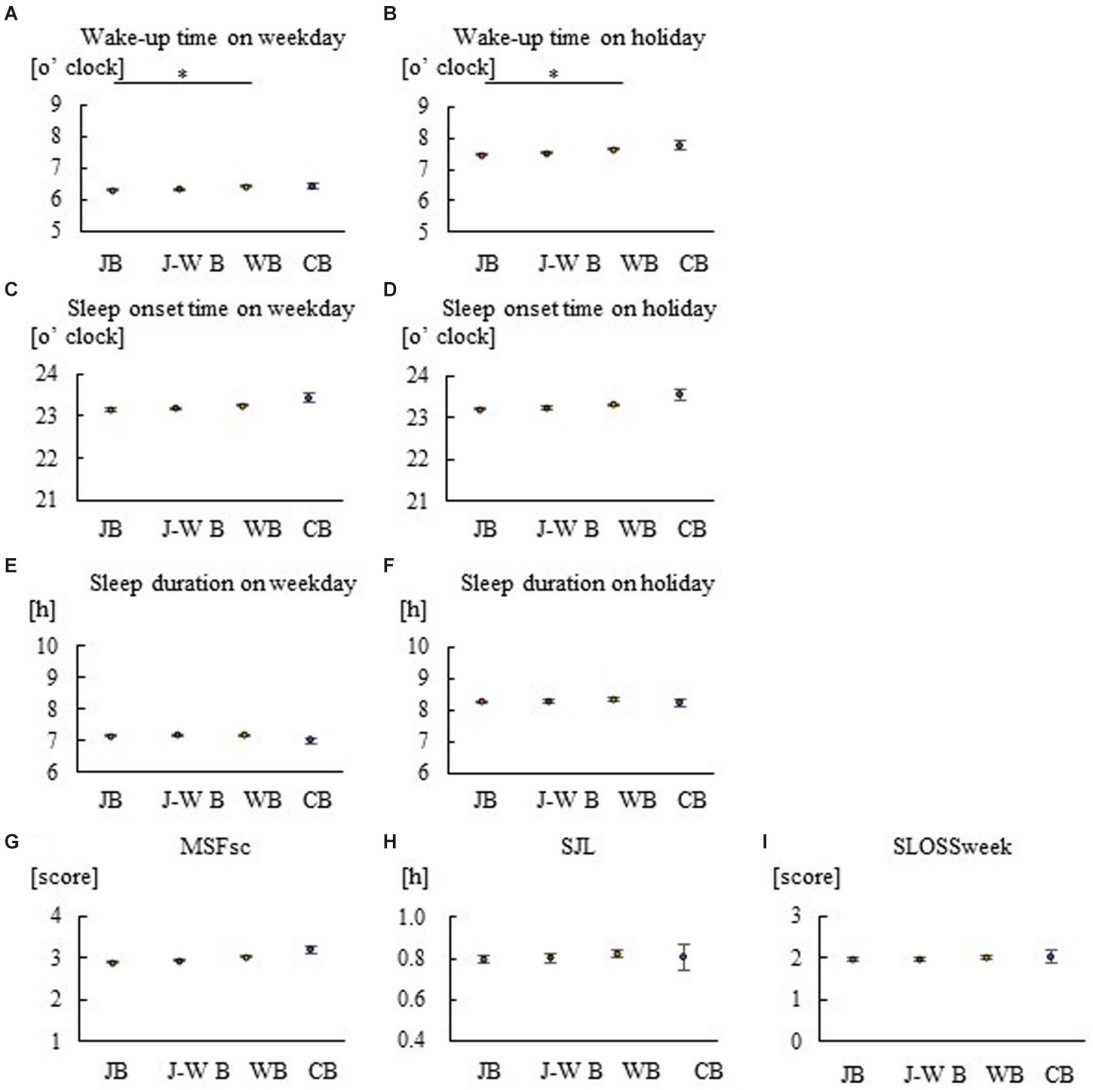
Group comparison of breakfast styles for sleep in fathers. Wake-up time on weekdays **(A)**, wake-up time on holidays **(B)**, sleep onset time on weekdays **(C)**, sleep onset time on holidays **(D)**, sleep duration on weekdays **(E)**, sleep duration on holidays **(F)**, MSFsc **(G)**, SJL **(H)**, and SLOSS **(I)**. Consumption of JB leads to earlier waking times compared with WB. *p < 0.05. A Kruskal–Wallis test with Bonferroni correction was used.

To examine the relationship between breakfast style and sleep habits, Spearman’s rank correlation coefficients were calculated using data for children, mothers, and fathers (Tables 2–4). Correlation analysis demonstrated that JB was negatively associated with sleep markers, including MSFsc, in children, mothers, and fathers, while WB and CB were positively associated. The correlation values in the JB groups were larger for mothers than for children and fathers. Comparisons were also made between breakfast-style groups regarding actual wakeup time, sleep onset time, sleep duration, and MSFsc, SJL, and SLOSS for children, mothers, and fathers (Figures 1–3). On both weekdays and holidays, families that consumed JB woke up earlier than those that consumed WB (Figures 1A, 2A, 3A). On both weekdays and holidays, the different styles arranged in increasing order of wake-up times was JB, J–WB, WB, and CB; this order was clearer and more significant on holidays (Figures 1A,B, 2A,B). The respective holiday wake-up time in the JB, WB, and CB groups for children were 7:12, 7:16, and 7:34; for mothers 6:52, 7:02, and 7:12; and for fathers 7:28, 7:38 and 7:45. Thus, the time difference between JB and WB was approximately 10 min and that between JB and CB was 20 min. Wake-up time was earlier for mothers, followed by children and fathers on both weekdays and holidays. Similar to the wake-up times, the increasing order of sleep onset time was JB, J–WB, WB, and CB (Figures 1C,D, 2C,D). On weekdays and holidays, children and their mothers who consumed JB went to bed earlier, while those who consumed WB or CB went to bed later. There were no significant differences in sleep duration between breakfast styles in children (Figures 1E,F) and fathers (Figures 3E,F). MSFsc values in the JB group were smaller among children (Figure 1G) and mothers (Figure 2G) than in the WB and CB groups, but not among fathers (Figure 3G). SJL values in the JB group were smaller among children (Figure 1H) and mothers (Figure 2H) than in the CB group, but not among fathers (Figure 3H). There were no significant differences in SLOSS among breakfast styles (Figures 1I, 2I, 3I). This suggests that consuming JB leads to earlier waking and sleeping times, more preference for morning hours, and less social jetlag. On the other hand, consuming WB or CB results in later waking and sleeping times, longer evening hours, more social jet lag, and worse sleep deprivation.

**Table 2:** Correlation between breakfast style and sleep in children.

	Wake-up time		Time of sleep onset		Sleep duration		MSFsc	SJL	SLOSS
	weekday	holiday	weekday	holiday	weekday	holiday			
JB	−0.04**	−0.04**	−0.03*	−0.04**	−0.00	0.00	−0.05**	−0.02	−0.01
J-WB	−0.02	−0.01	−0.02	−0.00	0.01	−0.01	−0.01	0.00	0.01
WB	0.04**	0.03*	0.02	0.02	0.01	0.02	0.03*	0.00	−0.00
CB	0.03**	0.06**	0.05**	0.06**	−0.03*	0.00	0.06**	0.04**	0.02

*p < 0.05, **p < 0.001. Spearman’s rank correlation coefficient was used.

**Table 3:** Correlation between breakfast style and sleep in mothers.

	Wake-up time		Time of sleep onset		Sleep duration		MSFsc	SJL	SLOSS
	weekday	holiday	weekday	holiday	weekday	holiday			
JB	−0.06**	−0.06**	−0.04**	−0.06**	0.01	0.01	−0.073**	−0.03*	0.01
J-WB	−0.03*	−0.02	−0.01	0.00	−0.01	−0.02	−0.00	0.02	0.00
WB	0.09**	0.06**	0.04**	0.05**	0.01	0.00	0.07**	−0.00	−0.02
CB	0.01	0.04**	0.04**	0.04**	−0.05**	−0.01	0.04**	0.04**	0.03*

*p < 0.05, **p < 0.001. Spearman’s rank correlation coefficient was used.

**Table 4:** Correlation between breakfast style and sleep in fathers.

	Wake-up time		Time of sleep onset		Sleep duration		MSFsc	SJL	SLOSS
	weekday	holiday	weekday	holiday	weekday	holiday			
JB	−0.04*	−0.05**	−0.03	−0.03	−0.00	−0.02	−0.03*	−0.02	−0.01
J-WB	−0.02	−0.01	−0.02	−0.01	0.01	−0.00	−0.01	0.01	−0.00
WB	0.04**	0.05**	0.03*	0.03	−0.00	0.02	0.04*	0.01	0.01
CB	0.01	0.02	0.02	0.03	−0.02	−0.01	0.03	0.00	0.01

*p < 0.05, **p < 0.001. Spearman’s rank correlation coefficient was used.

### 3.3. Relationship between breakfast style and frequency of food item intake

**Figure 4:**
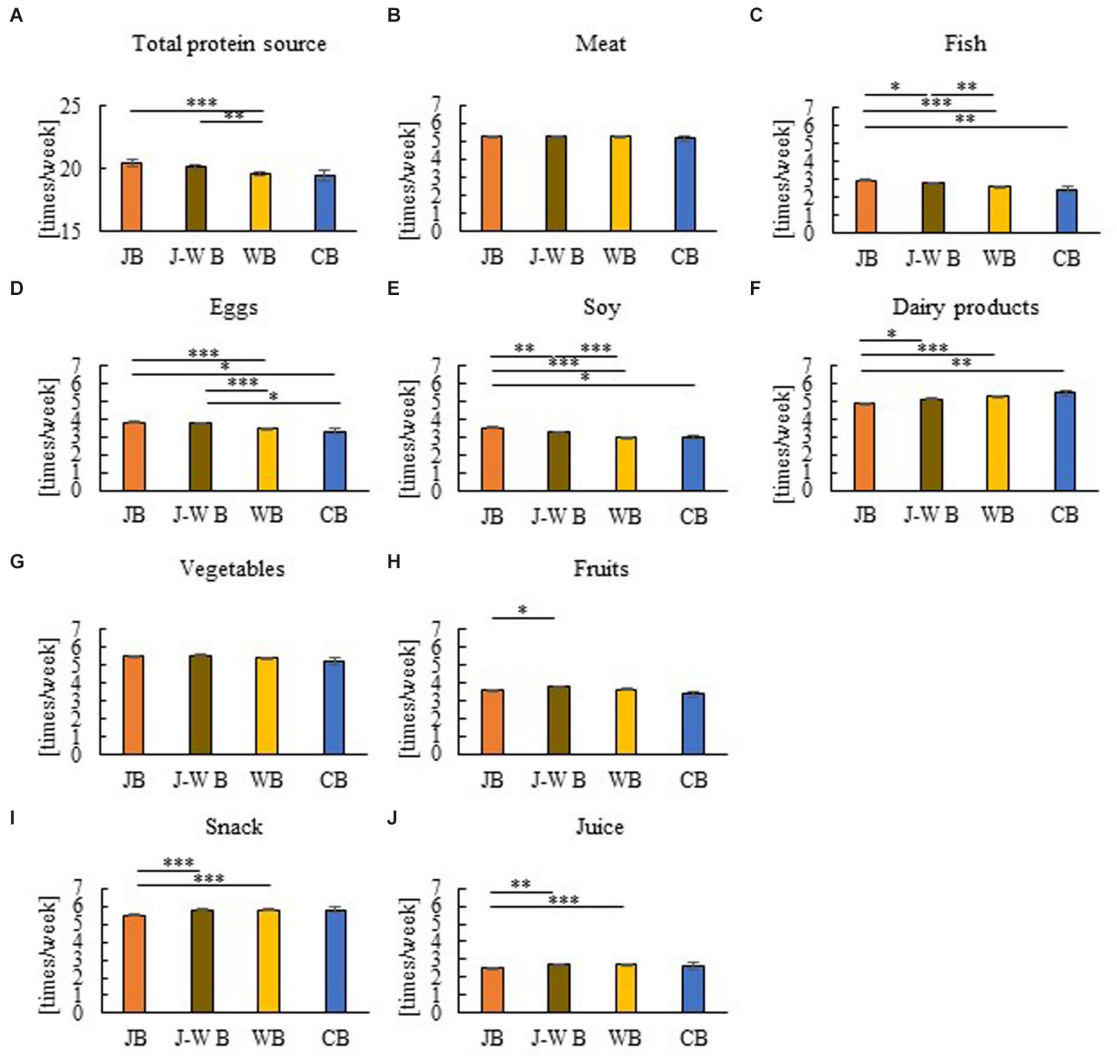
Group comparison of breakfast styles for frequency of food items in children. Total protein source **(A)**, Meat **(B)**, Fish **(C)**, Eggs **(D)**, Soy **(E)**, Dairy products **(F)**, Vegetables **(G)** Fruits **(H)**, Snack **(I)**, and Juice **(J)**. There is a link between the consumption of JB and the high intake of various protein sources (Fish, Eggs, and Soy), excluding dairy protein sources, and the low intake of snacks and juices. *p < 0.05, **p < 0.005, ***p < 0.001. A Kruskal–Wallis test with Bonferroni correction was used.

Spearman’s rank correlation coefficient was calculated for children’s data to examine the relationship between breakfast style and the intake frequency of various food items (Table 5). A comparison between breakfast-style groups was also made regarding children’s frequency of food item intake (Figure 4). The JB group was positively associated with fish, eggs, and soy and negatively associated with dairy products such as protein sources. In contrast, the WB and CB groups were negatively associated with fish, eggs, and soy and positively associated with dairy products. J–WB was positively associated with vegetables and fruits. JB was negatively correlated with WB snacks and juice intake frequency. Comparisons were also made between breakfast-style groups regarding the actual frequency (days/week) of food intake for children (Figure 4). There were no significant differences in meat intake frequency among breakfast styles (Figure 4B). Children who usually consumed JB had more protein sources such as fish, eggs, and soy than the WB or CB groups (Figures 4C–E). In contrast, the JB group had a significantly lower frequency of dairy products than the WB and CB groups (Figure 4F). There were almost no differences in vegetable and fruit intake frequency between the JB, WB, and CB groups (Figures 4G,H). The JB group had a lower intake of snacks and juices than the WB group (Figures 4I,J). There is a link between the consumption of JB and the high intake of various protein sources, excluding dairy protein sources, and the low intake of snacks and juices.

**Table 5:** Correlation between breakfast style and frequency of food items in children.

	Total protein source	Meat	Fish	Eggs	Soy	Dairy products	Vegetables	Fruits	Snack	Juice
JB	0.05^**^	0.01	0.08**	0.06**	0.10**	−0.07**	0.02	−0.02	−0.07**	−0.06**
J-W B	0.02	−0.00	0.01	0.04**	0.01	0.01	0.03*	0.04**	0.03*	0.02
WB	−0.05^**^	0.00	−0.08**	−0.08**	−0.11**	0.07**	−0.03	−0.00	0.06**	0.03**
CB	−0.02	−0.01	−0.03**	−0.03*	−0.02	0.03*	−0.02	−0.02	0.01	−0.00

*p < 0.05, **p < 0.001. Spearman’s rank correlation coefficient was used.

### 3.4. JB or WB characteristics with morning or evening type

**Figure 5:**
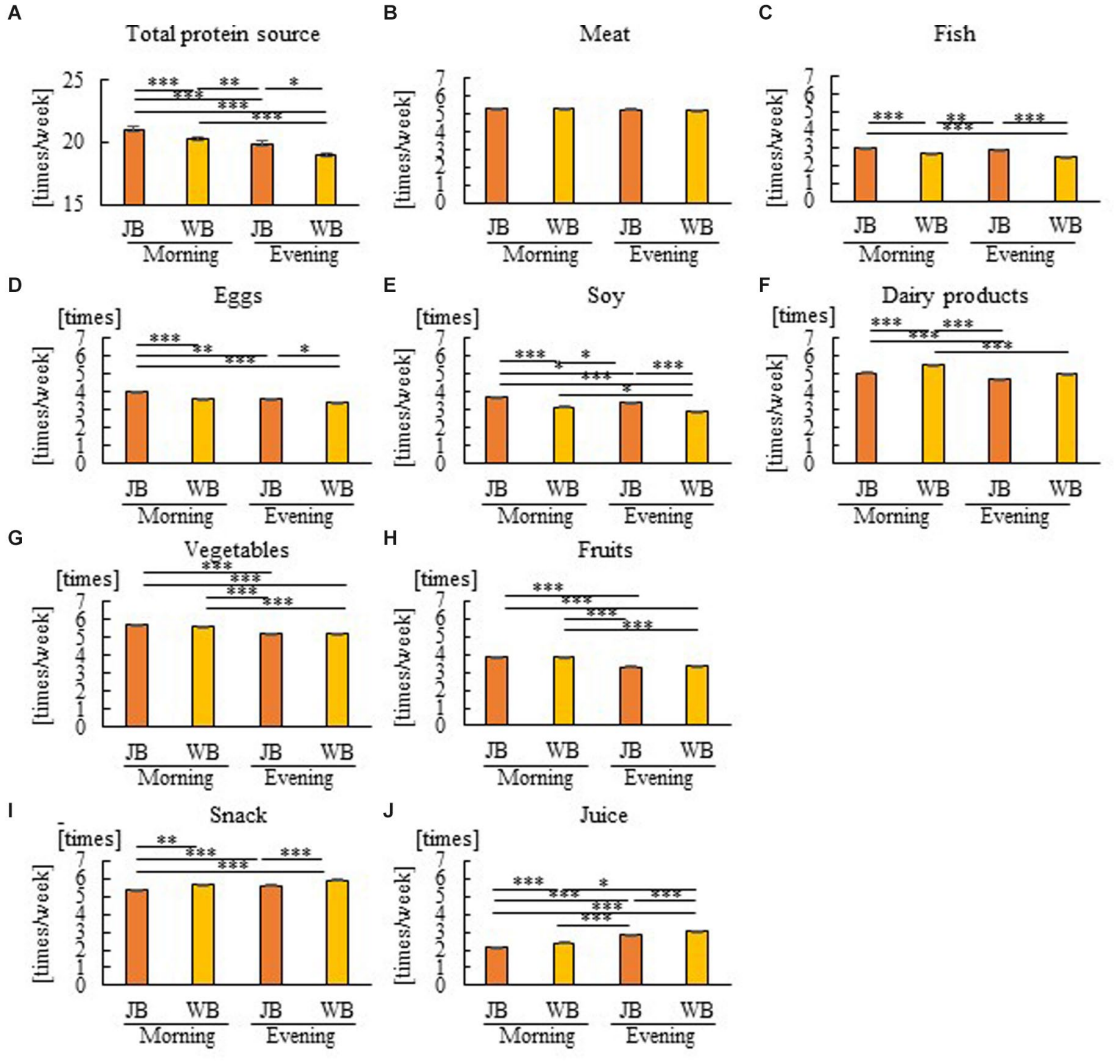
Group comparison of breakfast styles for frequency of food item in children between groups when divided into morning and evening types for JB and WB, respectively. Total protein sources **(A)**, Meat **(B)**, Fish **(C)**, Eggs **(D)**, Soy **(E)**, Dairy products **(F)**, Vegetables **(G)**, Fruits **(H)**, Snack **(I)**, and Juices **(J)**. JB groups with morning types exhibit healthy food consumption (Fish, Eggs, Soy, Vegetables, and Fruits). On the other hand, WB groups with evening types showed less consumption of healthy food and more consumption of unhealthy food (Snacks and Juice). * *p* < 0.05, ** *p* < 0.005, *** *p* < 0.001. A Kruskal–Wallis test with Bonferroni correction was used.

We recently reported that those who consume JB are more likely to be morning-type individuals, while those who consume WB are more likely to be evening type (27). Based on these results, we examined whether the intake of each food group was influenced by chronotype or by the difference between JB and WB. In the current experiments, the percentage of JB (36%) and WB (38%) was similar, and previous adult data (20–60 years old) showed a similar percentage of JB (30%) and WB (31%) (27). However, the percentages of J–WB (23%) and CB (3%) were lower in the present study; hence, we divided the JB and WB groups into morning and evening types. First, we divided the children into morning and evening types and further grouped them according to whether they consumed JB or WB. The characteristics and results of food intake are presented in Supplementary Table 4. The morning and evening type groups were each subdivided into JB and WB groups, and the food intake in each group was compared (Figure 5). As expected, the JB and WB groups among boys and girls were associated with the morning and evening types, respectively. The morning types had lower MSFsc values, while the evening type had higher ones. The mean intake frequencies of fish, eggs, and soy, were highest in the JB group with the morning types and lowest in the WB group with the evening type. The differences in mean values between the JB morning and EB evening types were 0.51, 0.50, and 0.84 for fish, egg, and soy intake, respectively. The differences in mean values between the JB morning and EB evening types were 0.57 and 0.80 for snacks and juice intake, respectively. There were no significant differences in meat intake among the four groups (Figure 5B). The frequency of food intake by fish (Figure 5C), total protein sources (Figure 5A), eggs (Figure 5D), soy (Figure 5E), vegetables (Figure 5F), and fruits (Figure 5G) were significantly higher in the JB group with the morning types than in the WB group with the evening type. The intake frequencies of snacks (Figure 5G) and juice (Figure 5H) were significantly lower in the JB group with the morning types than in the WB group with the evening types. In general, those who consumed JB with morning types exhibited healthy eating habits. On the other hand, those who consumed WB with evening types exhibited less consumption of healthy food.

### 3.5. Association of food consumption with MSFsc

The results of the multiple regression analysis showed that eating breakfast was associated with lower MSF. Multiple regression analysis also showed that the intake of soy, dairy products, vegetables, and fruits lowered MSF. On the other hand, the intake of meat, snacks, and juice increased MSF (Table 6).

**Table 6:** Multiple regression analysis of MSFsc with breakfast style and food consumption.

Target variable: MSFsc, adjustment factor: Age, Sex, BMI or POW								
	3–5 years				6–8 years			
	*β*	*p*	*R* ^2^	*F*	*β*	*p*	*R* ^2^	*F*
JB	−0.03	0.16	0.01	5.13	−0.04	0.03	0.02	11.84
J-W B	−0.02	0.27	0.01	4.95	−0.00	0.98	0.02	10.56
WB	0.02	0.37	0.01	4.84	0.02	0.21	0.01	10.96
CB	0.06	0.00	0.01	7.53	0.06	0.00	0.02	13.00
Total protein source	−0.15	0.00	0.03	21.7	−0.13	0.03	0.03	22.92
Meat	−0.03	0.10	0.01	5.32	0.00	0.82	0.02	10.57
Fish	−0.80	0.00	0.01	9.15	−0.05	0.01	0.02	12.40
Eggs	−0.10	0.00	0.02	12.13	−0.08	0.00	0.02	15.00
Soy	−0.14	0.00	0.03	19.20	−0.10	0.00	0.03	17.60
Dairy products	−0.11	0.00	0.02	12.83	−0.14	0.00	0.04	25.65
Vegetables	−0.12	0.00	0.02	15.87	−0.15	0.00	0.04	26.07
Fruits	−0.14	0.00	0.03	19.84	−0.12	0.00	0.03	20.43
Snack	0.10	0.00	0.02	11.24	0.05	0.00	0.02	12.18
Juice	0.18	0.00	0.04	27.50	0.15	0.00	0.04	27.70

*R*^2^ indicates the contribution ratio and *β* the standardized partial regression coefficient. BMI (Body Mass Index) is for 3–5 years and POW (Percentage of overweight) is for 6–8 years.

## 4. Discussion

To the best of our knowledge, this is the first cross-sectional study in Japan to examine the association between breakfast type, sleep, and eating habits among children aged 3–8 years. Our results showed that JB consumption was associated with good sleeping and eating habits.

### 4.1. Sleep and breakfast style

Adequate sleep from infancy onwards is important for development. However, many children have difficulties sleeping for the recommended duration (36). In this study, we found a close association between sleep and breakfast, which is consumed after long periods of starvation.

The National Sleep Foundation recommends 11–13 h of sleep for toddlers aged 3–5 years and 10–11 h for school-aged children aged 6–11 years (37, 38). The average duration of sleep is reported to be 10.4 h for toddlers aged 3–5 years and 8.9 h for school-aged children aged 6–11 years. Therefore, the average time of 9.47 h of sleep on weekdays and 9.83 h of sleep on weekends of participants in the current study was insufficient to meet the required sleep time. There were almost no differences in sleep duration on weekdays and holidays among breakfast styles (JB, J–WB, WB, and CB) in children, mothers, and fathers, suggesting that sleep duration is independent of breakfast style.

When compared with the WB and CB groups, the JB group showed an earlier pattern of wakeup and sleep onset times on weekdays and holidays, suggesting that children, mothers, and fathers who consume JB are more likely to be of the morning types rather than the evening. These findings are consistent with those of our recent studies on JB groups aged 20–60 years participating in body weight reduction programs (70% women) and JB groups aged 20–50 years participating in daytime work (70% men); individuals who often consume JB tend to be morning types when compared with the WB or CB groups (26, 27). The respective holiday wake-up times in the JB, WB, and CB groups for children were 7:12, 7:16, and 7:34; for mothers 6:52, 7:02, and 7:12; and for fathers 7:28, 7:38 and 7:45. Thus, the time difference between the JB and WB groups was approximately 10 min, and that between the JB and CB groups was 20 min. Every morning, 15 min of phase advance are required to adjust to a 24 h rhythm from circadian free running with 24.2–24.5 h (39). Thus, habitual changes in breakfast style from WB or CB to JB may move 10–20 min earlier than the circadian clock. The present study revealed that people with a pattern of consuming WB or CB tend to exhibit evening-type preference behaviors. The longer screen-time exposure by later sleep onset (40) may contribute to delaying the circadian clock time the next morning. CB and WB may further promote the evening type. Comparing children, mothers, and fathers, the results for mothers showed more differences in group comparisons and correlations with breakfast style. The order of wake-up time on weekdays and holidays was as follows: mothers, children, and fathers. This may be because mothers are often responsible for feeding their children breakfast in Japan, while many fathers are not much concerned about their children and lead their own lifestyles. It may take more time to prepare JB compared to WB or CB; therefore, JB may require earlier wake-up times on weekdays. However, the JB groups showed earlier wake-up times not only on weekdays, but also on holidays. The JB group had smaller SJL and better eating habits. There is a significant relationship between the number of times a child wakes up at night and the amount of sleep a mother gets; however, fathers are somewhat less affected by their children’s sleep disturbances (41), and it can be argued that a close relationship exists between children and mothers’ sleep. This suggests that mothers and children may influence each other regarding sleep. Moreover, insufficient sleep can lead to chronic diseases related to hypertension, diabetes, depression, and obesity, and reduced quality of life (42). An examination of the association between sleep and breakfast style revealed that consuming JB contributes to higher sleep scores. This is because people who can afford a JB tend to be early risers in the morning and tend to be morning types.

### 4.2. Effects of consuming Japanese food for breakfast on eating habits

Japanese food can provide an ideal nutritional balance (43, 44), and consuming Japanese food in the morning can result in a higher intake of protein sources such as fish, eggs, and soy. Since there is a relationship between consuming varying types of protein sources, excluding dairy protein sources, and JB, and since there is a relationship between increasing sleep scores and JB, there may also be a relationship between improving sleep scores and consuming protein sources.

Soy can prevent lipid-related diseases, including stroke and coronary heart disease (45). Furthermore, seafood prevents lifestyle-related diseases such as hypertension, diabetes, and lipid disorders. Furthermore, consuming JB was associated with a lower frequency of sweets and juice consumption. Consumption of high-calorie food and unhealthy snacks leads to obesity (46); therefore, consumption of Japanese food, which can reduce the frequency of sweets and juice intake, is directly linked to a healthy lifestyle. Soy and fruits are low-glycemic index (GI) foods (47). Consuming JB can lower total blood glucose levels during the day. In addition, Japanese food is rich in pulses, such as soy, which has a low GI. Eating low-GI foods in the morning improves the glycemic response and provides greater benefits than eating low-GI foods in the evening (48). Consuming Japanese food in the morning provides various protein sources and health benefits and controls blood glucose levels. In the future, analysis of studies over the years may help us understand the importance of breakfast style habits among preschool and elementary school children to protect against lifestyle-related diseases.

### 4.3. Relationship between morning-type tendencies, food intake, and health

In the present study, we evaluated the amount of food intake according to the time of day per week. Food frequency questionnaires are a popular way to estimate food intake; therefore, the present questionnaires may compare the amount of food consumed among different breakfast styles. The differences in mean values between JB with morning type and WB with evening type were 0.51, 0.50, and 0.84 for fish, egg, and soy intake, respectively. The differences in mean values between these two groups were 0.57 and 0.80 for snack and juice intake, respectively. These differences may lead to a 10% change in food intake. In general, a 10% change in food intake may be a factor for body weight, blood pressure, and metabolic changes. Similar results were obtained from the multiple regression analyses in the present study. Intake of soya, dairy products, vegetables, and fruit may contribute to changes in the morning types. Snacks and juice intake may contribute to the changes in the evening type.

In the present study, chronotypes, such as the morning or evening types, were found to influence food intake. The number of protein sources was significantly influenced by whether a person ate JB or WB, but was also influenced to a lesser extent by morning or evening types; individuals with morning type consumed more food. The results showed that the group that was morning-oriented and consumed JB usually consumed more protein sources, excluding dairy products. People who sleep late and eat WB, on the other hand, have fewer protein sources. The intake of vegetables, fruits, sweets, and soft drinks was also significantly influenced by whether they were morning or evening types. Compared to evening types, being a morning-type individual with JB is associated with a more active intake of vegetables and fruits. Furthermore, being an evening type individual with WB is associated with a more active intake of sweets and soft drinks.

Circadian rhythms influence diet (48). However, it is a new finding that morning-type individuals consume more vegetables and fruits, whereas evening-type ones consume more sweets and soft drinks. Furthermore, the circadian clock is involved in weight gain and obesity; being awake at night promotes obesity (48). In addition, vegetable and protein sources intake suppresses blood glucose levels and prevents obesity, while the consumption of sweets and soft drinks promotes obesity. In other words, being a morning-type individual prevents obesity and leads to good health through the consumption of vegetables and protein sources. Being a night owl increases the consumption of sweets and soft drinks, leading to obesity. Fruits also have the potential for cardiovascular protection, and reduced risk of colon cancer, depression, and pancreatic disease. Furthermore, vegetable intake reduces the likelihood of colon and rectal cancer, hip fracture, stroke, depression, and pancreatic disease (49). Reducing children’s consumption of energy-dense snacks and promoting healthier foods such as fruits and vegetables are effective and necessary means of improving their dietary intake and reducing their risk of chronic diseases later in life. Thus, our results strongly suggest that JB rather than WB or CB can be beneficial for evening-type children.

## 5. Limitations

Our study had certain limitations. First, food items and eating habits were collected through self-reports, and this might have resulted in self-efficacy. Second, although there are 50 characteristics of respondence, we used only three variables in this study (sex, age, and BMI). Therefore, more variables should be used to analyze the associations in future studies. Third, we did not include the age, BMI, and breakfast styles of mothers and fathers. Fourth, the association between sleep and eating habits should be assessed including not only the breakfast, but also the lunch and dinner styles to better understand the role of traditional Japanese food in healthy eating behavior. As a strength of the current study, the findings may be generalized to children in Japan, because we included participants without bias, such as people from cities and rural areas, and those from northern and southern areas of Japan.

## 6. Conclusion

Our results showed that JB may help develop morning-type habits regarding circadian rhythm and healthy eating habits. Earlier wake-up/sleep-onset times and eating habits established in infants may help prevent chronic diseases later in life.

## Data availability statement

The raw data supporting the conclusions of this article will be made available by the authors, without undue reservation.

## Ethics statement

The Ethics Review Committee on Research Involving Human Subjects at Waseda University approved this experiment (No. 2021-101). The guidelines of the Declaration of Helsinki were followed. The patients/participants provided their written informed consent to participate in this study.

## Author contributions

YT and SS: conceptualization and research ideas. LN, AF, YN, SM, and NM: methodology and data collection. MK and SS: data analysis, writing the original draft, and editing. SS: funding acquisition, writing the revised draft, and review. All authors contributed to the article and approved the submitted version.

## Funding

This research was funded by the Japan Society for the Promotion of Science (JSPS) KAKENHI (Kiban A) and JST-Mirai Program (Grant Number: JMPJM120D5).

## Conflict of interest

YN, SM, and NM were employed by Benesse Inc.

The remaining authors declare that the research was conducted in the absence of any commercial or financial relationships that could be construed as a potential conflict of interest.

## Publisher’s note

All claims expressed in this article are solely those of the authors and do not necessarily represent those of their affiliated organizations, or those of the publisher, the editors and the reviewers. Any product that may be evaluated in this article, or claim that may be made by its manufacturer, is not guaranteed or endorsed by the publisher.
