# Supplementary material for: Association of breakfast styles such as Japanese, Western, and cereals with sleeping habits, eating habits, and lifestyle in preschool and elementary school children
Source: Front Nutr. 2023 Jun 30;10:1131887. doi: 10.3389/fnut.2023.1131887 (PMC10348839; doi:10.3389/fnut.2023.1131887)
Supplement: Supplementary file 1 [file Data_Sheet_1.docx]

Supplementary Material

Association of breakfast styles such as Japanese, Western and Cereals on sleeping habits, eating habits and lifestyle in preschooler and elementary school children

Mai Kuwahara^1^, Yu Tahara^2^, Lyie Nitta^1^, Akiko Furutani^1^, Seiko Mochida^3^, Naomichi Makino^3^, Yuki Nozawa^3^, Shigenobu Shibata*^1^

*** Correspondence:** Shigenobu Shibata:shibatas@waseda.jp

# Supplementary Table

# Supplemental Table 1. Content of the question about breakfast style

| **Content of the question** | Which style of breakfast do you have? |
| --- | --- |
| **Breakfast category** | 1. JB, Japanese food (rice);  2. J-W B, Both Japanese and Western food (rice and bread);  3. WB, Western food (bread);  4. CB, cereals;  5. Skipping |

**Supplemental Table 2.**  Content of the question about weekly protein sources

| **Content of the question** | Have you consumed following protein rich food in the past four weeks? |
| --- | --- |
| **Score** | Score7; Almost every day;  Score4.5; 4-5 times per a week;  Score2.5; 2-3 times per a week;  Score1; Once per a week;  Score0; No; |
| **Food items** | Meat, Fish, Eggs, Beans (tofu, natto, etc.), Dairy products (milk, yoghurt, cheese, etc.) |

**Supplemental Table 3.**  Content of the question about weekly food items intake

| **Content of the question** | Have you consumed following food or drink in the past four weeks? |
| --- | --- |
| **Score** | Score7; Almost every day;  Score4.5; 4-5 times per a week;  Score2.5; 2-3 times per a week;  Score1; Once per a week;  Score0; No; |
| **Food items** | Vegetables, Fruits, Snacks, Juice (sugar-sweetened drink) |

**Supplemental Table 4.** Sleep information in children.

| **Category** | **ALL**  **(n=6104)** | **Boys**  **(n=3044)** | **Girls**  **(n=3060)** | **p Value**  **Boys vs. Girls** | |
| --- | --- | --- | --- | --- | --- |
| **Weekday wake-up time [hrs:min]** | 6:37 | 6:36 | 6:38 | 0.15 |  |
| **Holiday wake-up time [hrs:min]** | 7:14 | 7:09 | 7:19 | <0.001 |  |
| **Weekday sleep onset time [hrs:min]** | 21:09 | 21:09 | 21:09 | 0.68 |  |
| **Holiday sleep onset time [hrs:min]** | 21:25 | 21:24 | 21:25 | 0.37 |  |
| **Weekday sleep length [hrs:min]** | 9:27 | 9:27 | 9:28 | 0.48 |  |
| **Holiday sleep length [hrs:min]** | 9:49 | 9:45 | 9:53 | <0.001 |  |
| **Morning-Evening Type Index (MSFsc) [hrs:min]** | 2:08 | 2:07 | 2:10 | <0.005 |  |
| **Social Jet Lag (SJL) [hrs:min]** | 0:29 | 0:27 | 0:31 | <0.001 |  |
| **Sleep deprivation index (SLOSSweek) [hrs:min]** | 0:56 | 0:52 | 0:59 | <0.001 |  |

A Student’s t-test was used to analyze the data, which is expressed as the average ± SE.

**Supplemental Table 5.** Participants’ characteristics about eating habits in children

| **Category** | **ALL**  **(n=6,104)** | **Boys**  **(n=3,044)** | **Girls**  **(n=3,060)** | **p Value**  **Boys vs. Girls** |
| --- | --- | --- | --- | --- |
| **Total protein[times/week]** | 20.15±0.11 | 19.90±0.10 | 20.03±0.07 | p<0.05 |
| **Meat[times/week]** | 5.26±0.02 | 5.30±0.03 | 5.21±0.03 | p<0.05 |
| **Fish[times/week]** | 2.75±0.02 | 2.77±0.03 | 2.74±0.03 | 0.27 |
| **Eggs[times/week]** | 3.67±0.03 | 3.64±0.04 | 3.71±0.04 | 0.08 |
| **Soy[times/week]** | 3.27±0.02 | 3.28±0.04 | 3.26±0.03 | 0.31 |
| **Dairy products[times/week]** | 5.08±0.03 | 5.17±0.04 | 4.98±0.04 | p<0.001 |
| **Vegetables[times/week]** | 5.47±0.03 | 5.41±0.04 | 5.52±0.04 | p<0.05 |
| **Fruits[times/week]** | 3.63±0.03 | 3.61±0.04 | 3.64±0.04 | 0.33 |
| **Snacks[times/week]** | 5.68±0.02 | 5.62±0.03 | 5.74±0.03 | p<0.05 |
| **Juice[times/week]** | 2.65±0.03 | 2.69±0.04 | 2.60±0.04 | 0.07 |

A Student’s t-test was used to analyze the data, which is expressed as the average ± SE.

**Supplemental Table6.** Characteristics according to chronotype and breakfast style

| **Category** | **Morning　type** | | **Evening type** | |
| --- | --- | --- | --- | --- |
|  | **JB** | **WB** | **JB** | **WB** |
| Boys [people] | 575 | 505 | 564 | 618 |
| Girls [people] | 544 | 566 | 505 | 611 |
| Age [years] | 5.58±0.05 | 5.41±0.05 | 5.66±0.05 | 5.41±0.05 |
| MSFsc [o’clock] | 1:33 | 1:34 | 2:41 | 2:40 |
| Total protein sources [times/week] | 21.03±0.18 | 20.24±0.17 | 19.88±0.18 | 19.01±0.15 |
| Meat [times/week] | 5.30±0.05 | 5.29±0.05 | 5.26±0.05 | 5.24±0.04 |
| Fish [times/week] | 3.00±0.05 | 2.67±0.05 | 2.89±0.05 | 2.49±0.04 |
| Eggs [times/week] | 3.99±0.06 | 3.59±0.06 | 3.64±0.06 | 3.39±0.05 |
| Soy [times/week] | 3.70±0.06 | 3.16±0.06 | 3.4±0.06 | 2.86±0.05 |
| Dairy products[times/week] | 5.05±0.07 | 5.52±0.06 | 4.69±0.07 | 5.02±0.06 |
| Vegetables[times/week] | 5.77±0.06 | 5.66±0.06 | 5.2±0.07 | 5.22±0.06 |
| Fruits[times/week] | 3.82±0.07 | 3.91±0.07 | 3.30±0.07 | 3.37±0.06 |
| Snacks[times/week] | 5.35±0.06 | 5.68±0.06 | 5.62±0.06 | 5.92±0.05 |
| Juice[times/week] | 2.18±0.07 | 2.40±0.07 | 2.83±0.07 | 3.04±0.07 |

# A Student’s t-test was used to analyze the data, which is expressed as the average ± SE.
